# Epidemiological and phylogenetic characteristics of human metapneumovirus in Beijing, China, 2014–2024

**DOI:** 10.1038/s41392-025-02377-7

**Published:** 2025-09-09

**Authors:** Aihua Li, Cheng Gong, Liang Wang, Yuling Han, Lu Kang, Geng Hu, Jian Cao, Maozhong Li, Xuejiao Guan, Ming Luo, Lei Yu, Yuchuan Li, Fang Huang, George F. Gao, Quanyi Wang

**Affiliations:** 1 Beijing Key Laboratory of Surveillance, Early Warning and Pathogen Research on Emerging Infectious Diseases, Beijing Research Center for Respiratory Infectious Diseases, Public Health Emergency Management Innovation Center, Beijing Center for Disease Prevention and Control, Beijing, China; 2 School of Public Health, Capital Medical University, Beijing, China; 3 CAS Key Laboratory of Pathogen Microbiology and Immunology, Institute of Microbiology, Center for Influenza Research and Early-Warning (CASCIRE), CAS-TWAS Center of Excellence for Emerging Infectious Diseases (CEEID), Chinese Academy of Sciences (CAS), Beijing, China; 4 Children’s Hospital Affiliated to Capital Institute of Pediatrics, Beijing, China; 5 Beijing Children’s Hospital, Capital Medical University, National Center for Children’s Health, Beijing, China

**Keywords:** Molecular biology, Infection

## Abstract

In November 2024, there was an unusual surge in human metapneumovirus (hMPV) infection cases in Beijing. We performed an epidemiological investigation among cases with acute respiratory tract infection (ARTI). We enrolled ARTI cases from 35 sentinel hospitals, collected samples and medical records, conducted comprehensive pathogen testing, sequenced target genes or whole genomes, and performed phylogenetic analysis. A total of 79,793 cases were included in this study from 2014 to 2024. The hMPV epidemic exhibited typical seasonality from December to April of the following year, with an overall positivity rate of hMPV of 1.6%. The positivity rate of hMPV was highest in the 0–4 year age group (3.4%) and remained relatively high (1.2%) among populations over 60 years of age. Genotypes A and B were cocirculated, with predominant genotypes alternating every two years. We identified two variants of A2c with 180 or 111 nucleotide duplications in the G gene since 2016, and the A2c_111nt-dup_ has been predominant (56.9%) over the parent A2c since 2018. HMPV infection experienced an unusual surge beginning in November 2024 and peaked in December (9.5%). Subgenotype B2 (98.3%) returned to the predominant position instead of the A2c_111nt-dup_ and seemed to be associated with milder illness. Twenty hMPV isolates collected in 2024 were identified as known subgenotypes (A2c and B2) via whole-genome analyses. In conclusion, hMPV exhibited a typical seasonality in Beijing, with the predominant genotypes alternating every two years. Its unusual surge in November 2024 was attributed to the reoccurrence of hMPV B2 rather than a novel variant.

## Introduction

Since the beginning of the winter season of 2024–2025, ARTI cases have increased and have further surpassed the baseline level in some countries in the temperate Northern Hemisphere. A notable increase in human metapneumovirus (hMPV) infections has been observed in northern provinces of China, resulting in widespread concern (https://www.who.int/emergencies/disease-outbreak-news/item/2025-DON550).

HMPV, first identified in 2001, belongs to the *Metapneumovirus* genus within the family *Pneumoviridae*.^1^ It is considered a common respiratory virus responsible for the common cold, primarily affecting infants and children under five years of age. Annually, there are an estimated 14.2 and 0.0161 million hMPV-associated acute lower respiratory infection (ALRI) cases and deaths, respectively, among children younger than five years old.^2^ The clinical manifestations of hMPV infection are typically asymptomatic or mild in healthy adults, with symptoms such as cough, fever, sore throat, and runny nose.^3^ However, for high-risk populations, such as older people, immunocompromised individuals, and patients with underlying chronic conditions, hMPV infection can lead to severe clinical manifestations, such as pneumonia.^4^ Despite ongoing research and development, no approved antiviral drugs or licensed vaccines are currently available. Furthermore, hMPV is not routinely tested and monitored in many countries despite its substantial health burden, which is not conducive to global cooperation in response to infectious diseases.^5,6^

HMPV can be divided into two major genotypes, A and B, which are further categorized into six subgenotypes, A1, A2a, A2b, A2c, B1, and B2, on the basis of the sequence variation of the attachment (G) and fusion (F) glycoproteins.^7,8^ HMPV variants with 180- or 111-nucleotide duplications (nt-dup) in the G protein-encoded gene of A2c, designated A2c_180nt-dup_ and A2c_111nt-dup_ strains, were first described in Japan in 2014 and Spain in 2017, respectively.^9–11^ A2c_111nt-dup_ strains have been identified globally, such as in China, Croatia, and other countries, and are gradually becoming the dominant strains.^12–16^

The rise in hMPV infection cases has prompted concerns regarding the potential involvement of novel variants, thereby underscoring the critical need for sustained and ongoing surveillance of respiratory pathogens. This approach can also satisfy the needs for early alerts regarding emerging respiratory diseases that have the potential to cause epidemics or pandemics.^17,18^ Limited data are available regarding epidemiological and genetic features of hMPV in patients of all age categories in Beijing, particularly in the postcoronavirus disease (COVID-19) era. The Beijing Respiratory Pathogen Surveillance System (RPSS), a year-round regional surveillance system comprising 35 sentinel hospitals initiated in 2014, aims to monitor the epidemics of ARTI-associated respiratory pathogens. Here, we used surveillance data from this system to characterize the epidemiological features and genetic diversity of hMPV in Beijing between 2014 and 2024.

## Results

### Study population

Between September 1, 2014, and December 31, 2024, a total of 79,793 patients diagnosed with ARTI were investigated and sampled, spanning ten complete seasons (the 2014–2015 season to the 2024–2025 season). Male patients accounted for 54.9% (418 patients lacked sex information), which was consistent with the demographic characteristics in China. The age of the participants ranged from less than one month to 109 years, with a median age of 38 years (IQR: 8–66 years; 1691 patients were missing age information).

Among all included patients, hMPV was identified in 1245 patients (1.6%, 1245/79,793), and 514 (49.2%) patients were inpatients. With respect to clinical diagnoses, 460 (44.0%) patients had upper respiratory tract infections (URTI), 639 (61.1%) had non-severe community-acquired pneumonia (nsCAP), 97 (9.3%) had severe community-acquired pneumonia (sCAP), and 49 (4.7%) had other diseases (such as bronchitis and tonsillitis) (*P* < 0.05).

### Temporal dynamic changes in hMPV infection in Beijing

**Fig. 1:**
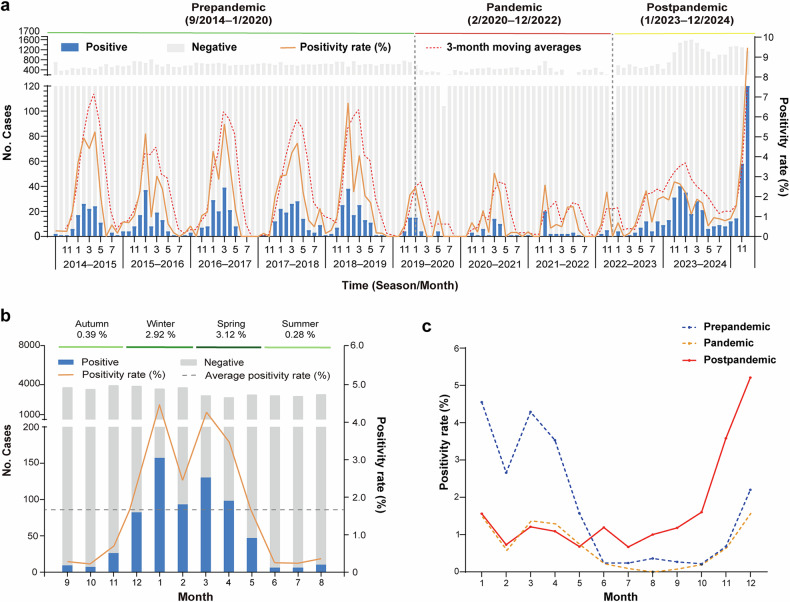
HMPV prevalence among all-aged patients with ARTI in Beijing, China, from September 1, 2014, to December 31, 2024. **a** The number of patients with ARTI and the positivity rates of hMPV infection by month. **b** Average percentage of positive detections for hMPV among patients with ARTI per month during the prepandemic era from September 1, 2014, to December 31, 2019. **c** Monthly average positivity rates of hMPV in the prepandemic era, pandemic era, and postpandemic era. The dashed lines (panel **b**) denote the threshold of the hMPV positivity rate (1.7%) assigned in this study

The hMPV epidemic among patients with ARTI in Beijing exhibits typical seasonality that spans from winter to spring. It usually increases significantly in December, peaks between January and March, and then declines after April of the following year. (Fig. 1a)

Prior to the onset of the COVID-19 pandemic, the overall positivity rate of hMPV infection was 1.7% (Fig. 1a). Owing to the notably low positivity rate of hMPV infection, we aggregated monthly positive case data from the five years preceding the COVID-19 pandemic. The epidemic season was subsequently identified on the basis of economic principles. Specifically, a month was designated as part of the epidemic season if its positive case count significantly increased the cumulative proportion of cases for that period relative to the annual total. This methodology allowed us to establish a monthly positivity rate of 1.7% as the threshold for the hMPV epidemic season, which successfully accounted for 82.7% of the total cases (Fig.1b). The average positivity rate of hMPV infection in both January and March during this period was greater than 4.0% (Fig. 1c).

During the COVID-19 pandemic, Beijing implemented strict public health management measures, leading to the transmission of hMPV at a low level. The overall positivity rate of hMPV was 0.7%. However, higher positivity rates were still observed from December to March of the following year than in other months (Fig. 1a, c).

In the postpandemic era following the COVID-19 outbreak, the overall positivity rate of hMPV was 1.8%. HMPV infections demonstrated a notable resurgence in June 2023, peaked in November 2023, and remained elevated until April of the following year. In 2024, hMPV was consistently detected throughout the year, with a notable increase in prevalence during the autumn season, reaching an unparalleled peak compared with that in the past decade (Fig. 1a). In addition, the peak of the hMPV epidemic in the postpandemic era occurred approximately one month earlier than that in the prepandemic era (Fig. 1c).

### Detection of hMPV in different populations

**Fig. 2:**
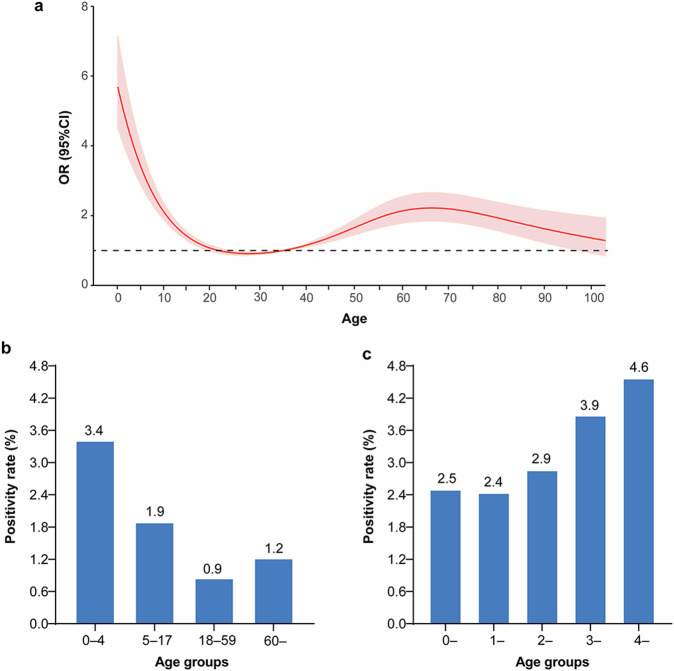
The positivity rate of hMPV among patients with ARTI by age in Beijing, China, from September 1, 2014, to December 31, 2024. **a** Analysis of the nonlinear influence of age on the risk of hMPV infection (*P* < 0.001). The rug plot along the x-axis shows the observed values; red shading indicates a 95% confidence interval (CI). Positivity rates among patients with ARTI grouped by age. **b** Positive hMPV among people of all ages. **c** Positive hMPV results in children under five years of age

A nonlinear association was observed between the hMPV infection risk and age. For patients under 25 years old, the hMPV infection risk decreased with increasing age. Conversely, among those aged 25 and older, the infection risk progressively rose with age, then gradually declined after the age of 65 (Fig. 2a).

Over the ten complete seasons, the positivity rates of hMPV varied by age, with the highest rate observed in children aged 0–4 years (3.4%), followed by a marked decline to 1.9% in those aged 5–17 years. The rate decreased to 0.8% in adults aged 18–59 years, and then increased slightly again to 1.2% in individuals aged 60 years or older (Fig. 2b). The distribution of positivity rates across different age groups in our study aligns with the trends identified through restricted cubic spline (RCS) analysis. For children aged <5 years, the highest positivity rate for hMPV was observed in the 4– < 5-year-old group (4.6%, 135/2954) (Fig. 2c).

### Coinfection of hMPV-positive patients

A total of 18.6% of the hMPV-positive patients (231/1245) were coinfected with at least one other respiratory virus. The most prevalent coinfection pathogen was seasonal influenza A (IFV A 22.1%), followed by respiratory syncytial virus (RSV, 19.5%), human rhinovirus (RV, 18.2%), *Mycoplasma pneumoniae* (MP, 11.7%), and parainfluenza virus (PIV: 1–4, 11.7%).

### Genotyping and phylogenetic analysis

Among the 1245 hMPV-positive samples, the F and G genes were amplified successfully in 64.8% (807/1245) and 39.9% (497/1245), respectively (Table S1). The remaining samples failed to be sequenced owing to low viral load or poor RNA quality. The obtained sequences were deposited in GenBank with accession numbers ranging from PV016180 to PV015672 for G gene, PV015673 to PV016467 for F gene. The reference sequences of each subgenotypes of hMPV are listed in Tables S2 and S3.

**Fig. 3:**
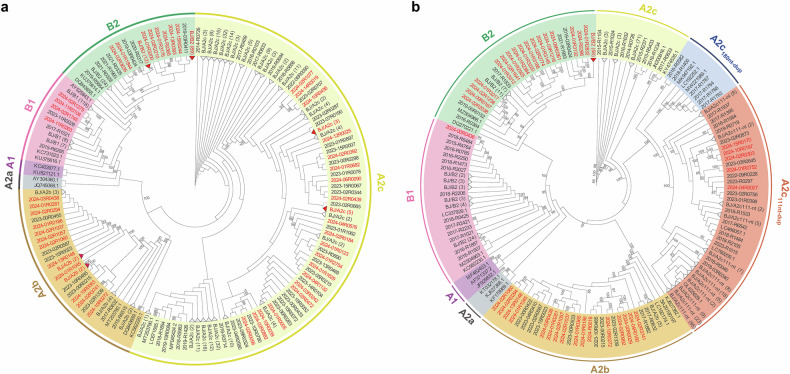
Phylogenetic analysis of the partial F gene (**a**) and G gene (**b**) of hMPV strains circulating in Beijing and the reference strains from GenBank. Different subgenotypes are labeled with different colors. The strain numbers in 2024 are highlighted in red font. Partial close strains from this study from 2014 to 2024 are represented by compressed black triangles, with the number of strains in brackets

Phylogenetic analysis of the F and G sequences revealed that the hMPV-A strains accounted for 48.0% (401/835), while hMPV-B strains accounted for 52.0% (434/835) (Fig. 3a, b). Overall, 37 (9.2%) samples belonged to A2b, 364 (90.8%) to A2c, 196 (45.2%) to B1, and 238 (54.8%) to B2. Subgenotypes A1 and A2a were not identified in the present study. Additionally, A2c_180nt-dup_ and A2c_111nt-dup_ variants were identified at frequencies of 1.2% (6/497) and 40.8% (203/497), respectively. Phylogenetic analysis revealed that A2c_111nt-dup_ and A2c_180nt-dup_ belong to different lineages (Fig. 3a, b).

### Circulation patterns of hMPV genotypes

**Fig. 4:**
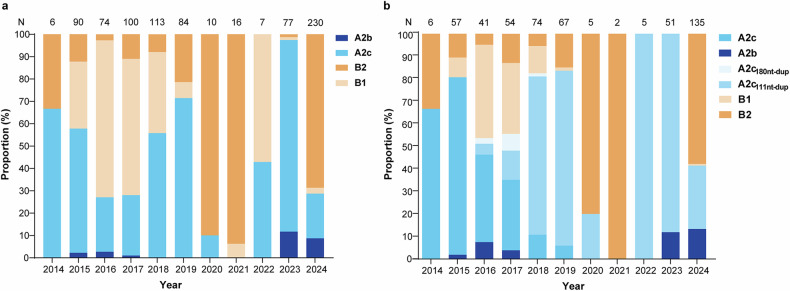
Circulation of the different subgenotypes of hMPV in the F gene (**a**) and G gene (**b**) from 2014 to 2024. N, the annual number of hMPV-positive specimens successfully sequenced

Between 2014 and 2024, both hMPV-A and hMPV-B cocirculated in Beijing. We define a genotype as belonging to season hMPV-A or hMPV-B if its proportion exceeds 50% according to the results of F gene sequencing. The predominant genotype changed every two years, indicating a possible switching pattern of “AABB” (Fig. 4a). A2b reappeared several years after its previous disappearance. The detection frequency of A2c_111nt-dup_ strains has gradually increased, and A2c_111nt-dup_ strains have emerged as the predominant strains since 2018. In contrast, only a small number of A2c_180nt-dup_ variants were detected between 2016 and 2018. Notably, the dominant subgenotype in 2023 was the A2c_111nt-dup_, whereas the dominant subgenotype changed to B2 in 2024. (Fig. 4b).

### Characteristics of hMPV prevalence in 2024

**Fig. 5:**
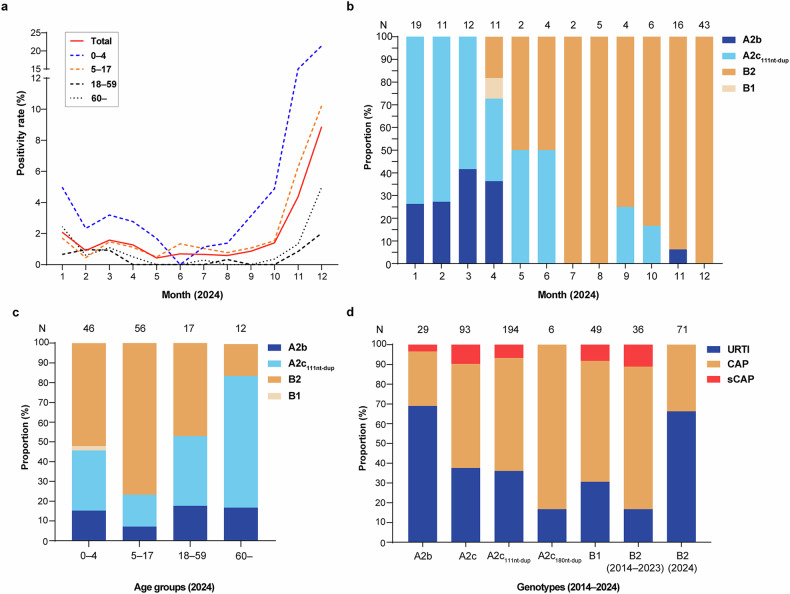
**a** Positivity rates of hMPV across the four age groups in 2024. **b** Monthly distribution of different subgenotypes in Beijing, January–December 2024. **c** Age distribution of different subgenotypes in Beijing, Jan–Dec 2024. **d** Distribution of clinical diagnoses according to the dominant genotypes in Beijing, 2014–2024. B2 represents subgenotype B2 in 2014–2023; B2 (2024) represents subgenotype B2 in 2024. URTI upper respiratory tract infection, nsCAP non-severe community-acquired pneumonia, sCAP severe community-acquired pneumonia. N, the total number of hMPV-positive specimens successfully sequenced

In 2024, a total of 17,961 patients with ARTI were enrolled and tested, yielding an overall positivity rate of 1.9% (343/17,961). In November, the positivity rate of hMPV increased significantly to 4.3% (58/1355) and increased markedly to 9.5% (120/1267) in December. In the winter of 2024, the positivity rate of hMPV was significantly higher compared to that during the corresponding period of the past nine years (6.8% vs. 1.6%) (Fig. 5a). In our study, outbreaks of hMPV infection in November and December 2024 predominantly affected children under 18 years of age (10.4%, 147/1411) (Fig. 5a).

Among the 343 positive cases, the G gene sequences were successfully obtained in 135 cases. From January to April 2024, the predominant subgenotypes were A2c_111nt-dup_ and A2b. Subgenotype B2 emerged in April and subsequently became the dominant subgenotype, replacing the A2c_111nt-dup_ by July (Fig. 5b). The number of cases attributed to B2 in 2024 significantly exceeded those caused by the A2c_111nt-dup_ and A2b (*P* < 0.05). Subgenotype B2 was detected mainly in people under 18 years of age (65.7%, 67/102), whereas A2c_111nt-dup_ was detected mainly in older adults over 60 years of age (66.7%, 8/12) (Fig. 5c). No cases of sCAP were identified in 2024 (Fig. 5d).

The most common symptom of hMPV infection is fever, followed by cough and rhinorrhea. No statistically significant differences were observed in the clinical symptoms among the subgenotypes, including B2 in 2024 and B2 in previous years (*P* > 0.05). The proportion of inpatients and the proportion of CAPs for B2 in 2024 was significantly lower than those of other subgenotypes (*P* < 0.05) (Table 1).

**Table 1:** Clinical symptoms of hMPV-positive patientes among the predominant genotypes in Beijing, 2014–2024

Symptoms and signs, *n* (%)	A2c	A2c_111nt-dup_	B2	B2(2024)	*P* value
	(*n* = 94)	(*n* = 190)	(*n* = 31)	(*n* = 63)	
Fever (≥37.3 °C)	82/92 (89.1)	170/177 (96.0)	28/29 (96.6)	59/61 (96.7)	0.719
<37.3	10/92 (10.9)	7/177 (39.5)	1/29 (3.4)	2/61 (3.3)	0.349
37.3–37.9	7/92(7.6)	15/177 (8.5)	0 (0)	2/61 (3.3)	
38–38.9	42/92 (45.7)	87/177 (49.2)	14/29 (48.3)	30/61(49.2)	
≥39	33/92 (35.9)	68/177(38.4)	14/29 (48.3)	27 (44.3)	
Sore throat	18 (19.1)	50 (26.3)	10 (32.3)	17 (27.0)	0.525
Cough	90 (95.7)	171 (90.0)	30 (96.8)	57 (90.5)	0.343
Nasal congestion	15 (16.0)	49 (25.8)	9 (29.0)	19 (30.2)	0.494
Rhinorrhea	46 (48.9)	68 (35.8)	15 (48.4)	28 (44.4)	0.277
Headache	1 (1.1)	9 (4.7)	4 (12.9)	3 (4.8)	0.191
Fatigue	4 (4.3)	15 (7.9)	5 (16.1)	1 (1.6)	0.133
Myalgia	0 (0)	12 (6.3)	1 (3.2)	1 (1.6)	0.057
Diarrhea	3 (3.2)	3 (1.6)	2 (6.5)	1 (1.6)	0.282
Abdominal pain	2 (2.1)	4 (2.1)	2 (6.5)	5 (7.9)	0.281
Disease severity status					
URTI	35/92 (38.0)	64/180 (35.6)	6/30 (20.0)	50/62 (80.6)	0.000
nsCAP	48/92 (52.2)	104/180 (57.8)	21/30 (70.0)	12/62 (19.4)	
sCAP	9/92 (9.8)	12/180 (6.6)	3/30 (10.0)	0 (0)	
Inpatient	45 (47.9)	73 (38.4)	14 (45.2)	5 (7.9)	0.000
ICU admission	2 (2.1)	6 (3.2)	1 (3.2)	1 (1.6)	0.881
Death in hospital	0 (0)	0 (0)	1(3.2)	0 (0)	0.000
Hospital length of stay (d)	8 (6,12)	8 (5,12)	7 (16,8)	8 (6,11)	0.229
Time from illness onset to discharge (d)	12 (11,16)	12 (9,15)	11 (9,12)	8 (6,11)	0.187

Data are n (%) or median (IQR). The data presented in the table encompass the cases with clinical information among the hMPV-positive samples that underwent successful G gene sequencing. These samples were collected in Beijing during the period from 2014 to 2024. B2 represents subgenotype B2 in 2014–2023. B2 (2024) represents subgenotype B2 in 2024. *P* values were calculated by Kruskal-Wallis test, χ 2 test, or Fisher’s exact test, as appropriate

**Fig. 6:**
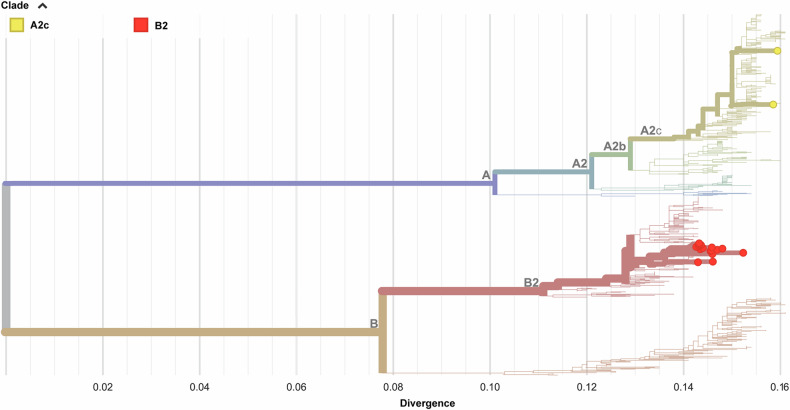
Phylogenetic tree of the complete hMPV genomes identified in this study. The phylogenetic tree was visualized via an online tool (https://www.auspice.us/). The tips with circles indicate the genomic sequences generated in this study. The other sequences are reference sequences. The color of the circle corresponds to different clades

We successfully obtained 20 hMPV genome sequences, and no novel subgenotype was identified. Eighteen hMPV-positive cases detected between November and December 2024 were identified as subgenotype B2 (Fig. 6). The sequences were deposited in GenBank, and the accession numbers were listed in Table S4.

**Fig. 7:**
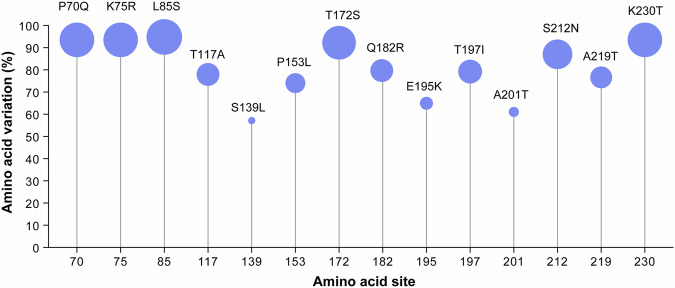
High-frequency mutation amino acids in the G protein of subgenotype B2 sequences in 2024 compared with genotype B2 sequences in 2014–2023. In 2024, a total of 72 subgenotype B2 sequences were identified, with the percentage indicating the proportion of sequences that contained amino acid site mutations

Analysis of the amino acid sequences derived from the G protein from subgenotype B2 samples collected in 2024, compared with those from 2014 to 2023, revealed that all the observed variations were base substitutions, with no deletions, insertions, or frameshift mutations identified. Using the B2 prototype strain NL/1/94 (GenBank accession no. AY296040) as the reference, the following high-frequency substitutions (proportion >50%) were identified in 2024: P70Q, K75R, L85S, T117A, S139L, P153L, T172S, Q182R, E195K, T197I, A201T, S212N, A219T, and K230T (Fig. 7).

## Discussion

HMPV is a common pathogen of ARTI with a much lower prevalence in China than other respiratory pathogens, such as the influenza virus or MP.^14,19–21^ This study was based on the RPSS in Beijing and included 10 years of continuous surveillance of the prevalence of hMPV in Beijing. ARTI associated with hMPV have increased since November 2024 in northern provinces of China (https://www.chinacdc.cn/jksj/jksj04_14275/202412/t20241205_303113.html). We also detected a concurrent increase in hMPV prevalence in Beijing, which was initiated in November. Given that Beijing is a megacity with a population of 21 million, the results of this study could be treated as a snapshot of hMPV epidemiology in northern China.

HMPV infection exhibited distinct seasonality in Beijing, with the epidemic period occurring from December to April and peaking between January and March. This was consistent with the hMPV epidemic pattern reported by previous studies in Beijing,^22–24^ as well as with trends observed in other countries within the temperate Northern Hemisphere.^25^ The overall positivity rate of hMPV infection was 1.6% (1245/79,793), which was lower than that reported in a nationwide Chinese surveillance study of ARTI across all age groups (4.1%).^26^ This difference was probably attributed to variations in the scope of the studies and local climatic conditions. Interestingly, a holiday effect linked to the Chinese Spring Festival was noted. The positivity rate of hMPV showed a slightly decline during this period, which was hypothesized to be related to the winter vacation for kindergarten-aged and school-aged children. During the winter vacation, the frequency of close contact among children decreased significantly. A similar trend was observed in our epidemiological surveillance of other viruses, such as varicella-zoster virus (VZV), whose transmission dynamics also correlate with school holiday periods.^27^ The prevalence of hMPV exhibited distinct fluctuation throughout the entire study period. The positivity rate was significantly lower during the COVID-19 pandemic era than in the prepandemic era (0.7% vs. 1.7%). A decrease in the prevalence of multiple respiratory pathogens, including IFV and MP, was also observed during the COVID-19 pandemic era. This decline was probably attributed to the nonpharmaceutical intervention (NPI) measures adopted against COVID-19.^28^ However, the resurgence of hMPV was observed following the relaxation of social distancing measures. From January to April 2023, surveillance data from the USA National Respiratory and Enteric Virus Surveillance System (NREVSS) (https://www.cdc.gov/nrevss/php/dashboard/index.html) revealed a significant peak in hMPV cases, which then returned to normal levels. In our research, following the suppression of hMPV circulation, an unusual resurgence occurred during the off-season and has persisted since June 2023. Moreover, our data also demonstrated a distinct pattern of year-round transmission in 2024, with a substantial increase in cases in November and an unprecedented peak in December. Similar prevalence patterns have also been reported in countries such as Israel, Australia, Spain, and South Korea, with an outbreak of hMPV occurring during the non-endemic season, and a shift to earlier time for the epidemic season. These observations may be attributed to the accumulation of susceptible individuals and a concomitant decline in population immunity resulting from the prolonged implementation of NPIs.^29–32^ This alteration also means that predicting the prevalence of hMPV in Beijing over the coming months will remain challengeable. In addition, we obtained another interesting finding in this study by incorporating our previous research: hMPV epidemics appear to consistently occur in synchrony with or slightly lag behind the RSV epidemic season.^33^ This phenomenon was evident not only during their typical epidemic seasons, which were generally in winter and spring each year but also in their out-of-season epidemics, which were observed in May and June 2023 following the increase in NPIs related to the COVID-19 pandemic.^33^ We speculated that this synchronization in terms of epidemic seasonality was associated with their close genetic distance and similar vulnerable population. The synchronization of the epidemic season has also increased the likelihood of coinfection with hMPV and RSV.

We found that the population vulnerable to hMPV presented distinct characteristics similar to those of the RSV population.^34^ HMPV infections affect almost all individuals in all age categories. This finding was consistent with a seroprevalence analysis of hMPV infections in Beijing, which suggested that all children over the age of six years were exposed to hMPV infection.^35^ We found that children under five years of age exhibited the highest positivity rate among all age groups. Our research findings are in alignment with those of previous studies.^26,36,37^ The positivity rate of hMPV in elderly individuals over 60 years of age was second only to that in children, which revealed that surveillance and prevention of hMPV among elderly individuals are as critical as those in children. Notably, among children under five years of age, the infection rate of hMPV increased with age and peaked at 3–4 years of age in our study, which was different from that of RSV: RSV predominantly infects children under two years of age. Xie. et al. reported a similar conclusion.^14^ This difference is worthy of further research and contributes to vaccination policy in the future.

In this study, hMPV-A and hMPV-B exhibited alternating cycles of predominance, following a pattern referred to the “AABB” pattern. In addition to subgenotypes A1 and A2a, four subgenotypes of hMPV (A2b, A2c, B1, and B2) were detected in Beijing, with two significant subgenotype shifts. We identified two kinds of variants, A2c_180nt-dup_ and A2c_111nt-dup_, which were inserted by 180 nucleotides and 111 nucleotides in G genes, respectively, and have been widely reported both globally and across China.^12–14,21,38^ These similar mutations are also found in RSV and contribute to evasion of the immune response by altering the glycosylation of the G protein.^39^ A2c_180nt-dup_ variants constituted a minor proportion of total hMPV infections and prevailed for only three years in Beijing. In contrast, A2c_111nt-dup_ variants have increased annually since their emergence in 2016 and became the predominant subgenotype from 2018 onward, which was the first subgenotype shift we observed. A similar subgenotype shift was reported in Japan in the corresponding period.^16^ The second major subgenotype shift occurred in 2024. Starting in April, subgenotype B2 re-emerged and subsequently replaced the A2c_111nt-dup_ as the dominant subgenotype by July. Subgenotype shifts in hMPV have also been reported in several countries, such as Israel, Western Australia, Spain, and South Korea, during the post-COVID-19 period, with higher prevalence rates of hMPV than in the prepandemic era, highlighting the importance of increased attention to subgenotype switching in hMPV.^29–32^

The exact reasons for the increased prevalence of hMPV during the autumn and winter of 2024 remain unclear. This study explored this issue from two perspectives. First, 2024 coincided with the expected transition from hMPV-A to hMPV-B, in accordance with the established “AABB” epidemic pattern. Second, the decreased prevalence of hMPV during the COVID-19 pandemic, attributable to NPIs, has resulted in diminished cross-protective immunity among the population against predominant subgenotype strains of hMPV. Therefore, we inferred that the synergistic effect of the two factors mentioned above elevated the hMPV prevalence in 2024. We did not observe distinct clinical manifestations of hMPV infection that could effectively differentiate hMPV from other respiratory pathogens (e.g., CoV and RSV) in the present study. The most common symptom of hMPV infection was fever, followed by cough and rhinorrhea. We further compared the clinical manifestations among different subgenotypes, including the recurring B2 and the previous B2, but found no significant differences among them. Specifically, the current hMPV epidemic, which was initiated in November 2024, presents clinical characteristics that are analogous to those observed in prior outbreaks. Moreover, both the proportion of inpatients and the proportion of CAP for the reoccurring subgenotype B2 were significantly lower than those for the other subgenotypes, including the previous subgenotype B2, which suggested that the current hMPV infection caused by the reoccurring subgenotype B2 seemed to be relatively mild compared with previous hMPV infections. We performed whole-genome analyses and further confirmed that the genomes obtained in this study were confirmed to belong to known subgenotypes. In conjunction with other evidence from this study, the increase in hMPV cases in Beijing could not be attributed to the emergence of a novel subgenotype of hMPV. The G protein of hMPV is highly glycosylated and variable, particularly the extracellular domain.^40^ In the present study, we identified 14 high-frequency amino acid substitutions in the extracellular region of the G protein of subgenotype B2 strains isolated in 2024 compared with earlier B2 strains. This region contains the receptor-binding domain and numerous potential protective antigenic epitopes,^41,42^ suggesting that these substitutions may have altered viral pathogenicity and contributed to immune evasion. These high-frequency mutations may be associated with the increased prevalence of the epidemic and the milder clinical manifestations observed in 2024.

The retrospective nature of this study represents a limitation in that limited genomic data were obtained in this study. Future research will aim to expand the number of genomes analyzed to reflect evolutionary relationships more comprehensively. However, this limitation did not affect the reliability of the main findings of this study.

In conclusion, hMPV prevalence exhibited a distinct “AABB” pattern, characterized by an annual epidemic season typically occurring between December and April. Children under five years of age were the major susceptible population, and attention should also be paid to the elderly over 60 years old. HMPV is a well-recognized respiratory pathogen, and its positivity rate is expected to exhibit seasonal variations. The surge did not indicate an emerging public health crisis but rather a pattern consistent with historical trends. In addition, the unprecedented hMPV prevalence detected in 2024 was caused by the reoccurrence of B2 rather than a novel variant. Therefore, there is no cause for public concern. Our findings provide comprehensive insight into hMPV prevalence in Beijing, even in northern China.

## Population and methods

### Ethics statement

This study was approved by the Ethics Committee at the Beijing Center for Disease Prevention and Control (BJCDC). Written informed consent was procured from each participant or their guardians (when the participant’s age or health status precluded direct consent) following a comprehensive explanation of the study’s nature, purpose, procedures, and potential health implications.

### Study design

This study was conducted based on the RPSS network, spanning the period from September 1, 2014, to December 31, 2024.

Patients across all age groups who were diagnosed with URTI or CAP during either outpatient or inpatient visits and who had attended any of the hospitals were eligible for inclusion in the study. URTI was defined as fever with a body temperature of ≥38 °C, with or without respiratory signs or symptoms, such as cough or sore throat, nasal congestion, rhinorrhea or sputum production). The definition of CAP follows the diagnostic and therapeutic guidelines issued in China^43–45^ and was further categorized into CAP or nsCAP.^46^

All patients were interviewed by their physicians upon enrollment, employing a standardized questionnaire. The questionnaires gathered information regarding demographic data, epidemiological details, and clinical presentations. The respiratory specimens was collected upon presentation for outpatients or within 72 h following hospital admission. The samples were transferred to the collaborating laboratories for the detection of respiratory pathogens by multiplex combined real-time reverse transcription PCR (multiple RT-PCR).

In Beijing, the hMPV activity was increased during December and April of the following year.^22^ To better compare the changes in hMPV epidemic timing between different years, we select the period from September 1 through August 31 of the next year to observe yearly epidemics of hMPV.

### Laboratory examination

Total nucleic acids were extracted from the samples via Thermo Scientific^TM^ KingFisher^TM^ Flex Magnetic Particle Processors (Thermo Fisher). A commercial multiple RT-PCR detection kit was employed to identify respiratory viruses^46^ (Jiangsu Uninovo Biological Technology Co. Ltd., China), including IFV (A-H1N1, A-H3N2, and B), PIV (1–4), RSV, RV, human adenovirus (AdV), human bocavirus (BoV), hMPV, MP, human coronavirus (CoV: NL63, OC43, 229E, and HKU1), enterovirus (EV), and SARS-CoV-2.

### HMPV genotyping and sequencing

For all the hMPV RT-PCR-positive samples, the partial sequences of the F and G genes were amplified via a one-step RT-PCR kit (Invitrogen). The primers used for G gene^9^ amplification were as follows: hMPV-SH7: 5′-TACAAAACAAGAACATGGGACAAG-3′ and hMPV-GR: 5′-GAGATAGACATTAACAGTGGATTC-3′. The primers for the F gene^13^ were as follows: hMPV-F-F: 5′-CAATGCAGGTATAACACCAGCAATATC-3′ and hMPV-F-R: 5′-GCAACAATTGAACTGATCTTCAGGAAAC-3′. All RT-PCRs were composed of 5 μL of RNA, 1 μL of each forward and reverse primer (10 μM), 1 μL of SuperScript^TM^ III RT/Platinum^TM^ Taq Mix, and 12.5 μL of 2 × Reaction Mix, with a final volume of 25 μL supplemented with distilled water. The thermal cycle conditions were as follows: reverse transcription at 50 °C for 30 min, holding at 94 °C for 2 min, 40 cycles of denaturation at 94 °C for 30 s, annealing at 58 °C (for the G gene)/56 °C (for the F gene) for 30 s, and extension at 72 °C for 1 min, followed by a final extension at 72 °C for 10 min. The amplicons were analyzed via electrophoresis in a 2% agarose gel. The PCR products were purified and sequenced via an ABI3730xl DNA Analyzer at Sangon Biotech (Shanghai, China).

### Metatranscriptomic and hybrid capture-based sequencing

Thirteen microliters of RNA were used for library preparation (IGT™ Fast Stranded RNA Library Prep Kit v2.0). An aliquot of the 750 ng library from each sample was used for hybrid capture-based enrichment of hMPV with one or two rounds of hybridization (TargetSeq One®, Hyb & Wash Kit v2.0).^47^ Sequencing was performed on the Illumina HiSeq X Ten platform. Whole-genome assembly: Quality control was executed via fastp v0.20.0 (2016).^48^ This process entailed the trimming of adapters, as well as the removal of low-quality and short reads. The remaining clean reads were then de novo assembled via MEGAHIT v1.2.9.^49^ To identify the hMPV genomes accurately, the assembled contigs were compared with hMPV reference genomes retrieved from the NCBI database via BLAST. For each sample, the genome with the highest sequence identity was chosen as the reference. Finally, the complete hMPV genomes were refined and generated via iVar-1.3.1.^50^ The number of reads for the samples were provided in Table S4. The read lengths are uniformly distributed within the range of 1-150 base pairs.

### Phylogenetic analysis

The SeqMan program (DNASTAR, Inc., Madison, WI) was used for contig assembly and for obtaining the G/F gene. The partial and/or complete hMPV G/F gene nucleotide sequences were aligned via ClustalW in MEGA 6.0. Maximum-likelihood (ML) phylogenetic trees were generated and tested via IQ-TREE v2.0.3^51^ via the bootstrap method with 1000 replications. For the hMPV genomes obtained from this study, we used Nextclade version 3.9.1^52^ to perform genomic alignment and phylogenetic tree reconstruction.

### Analysis of the deduced amino acid sequence

To analyze potential amino acid (aa) variations, we conducted a comparative sequence analysis of the hMPV isolates obtained in our study against the reference sequence of the prototype B2 strain (NL/1/94) in BioEdit 7.0.9.

### Statistical analysis

Continuous variables are summarized as medians (interquartile ranges, IQRs) and were compared across groups via the Kruskal-Wallis test; categorical variables are presented as numbers (%), and were compared between groups using the χ2 test or Fisher’s exact test, as appropriate. Statistical significance was defined as a two-sided α < 0.05. Cases with missing values for a given variable were excluded from its respective analysis. All the data were entered independently by two technicians using EpiData 3.1 (Odense, Denmark). All the statistical analyses were performed via SPSS 23.0 software. The RCS method was employed to examine the association between the risk of hMPV infection and age using R 4.0.5 and the RMS software package.

## Supplementary information

Supplementary Material

## Data Availability

The data supporting the conclusions of this article are available within the article and/or its Supplementary Materials. Further data will be provided by the corresponding author upon reasonable request.
