# Supplementary material for: Epidemiological and phylogenetic characteristics of human metapneumovirus in Beijing, China, 2014–2024
Source: Signal Transduct Target Ther. 2025 Sep 9;10:300. doi: 10.1038/s41392-025-02377-7 (PMC12417542; doi:10.1038/s41392-025-02377-7)
Supplement: Supplementary file 1 — Supplementary Material [file 41392_2025_2377_MOESM1_ESM.doc]

Supplementary Materials for

Epidemiological and phylogenetic characteristics of human metapneumovirus in Beijing, China, 2014–2024

Aihua Li^1,2^, Cheng Gong^1,2^, Liang Wang^3^, Yuling Han^1,2^, Lu Kang^1,2^, Geng Hu^1,2^, Jian Cao^3^, Maozhong Li^1,2^, Xuejiao Guan^1,2^, Ming Luo^1,2^, Lei Yu^4^, Yuchuan Li^5^, Fang Huang^1,2^, George F. Gao^3*^, Quanyi Wang^1,2*^

Correspondence to: Quanyi Wang [(wangqy@bjcdc.org)](mailto:(wangqy@bjcdc.org))

**This PDF file includes:**

Tables S1-S4

**Table S1. The number of successful sequences for the F and G genes in the samples from confirmed positive cases over the years.**

| year | positive cases | sequences of G gene | sequencing rate(%) | sequences of F gene | sequencing rate(%) |
| --- | --- | --- | --- | --- | --- |
| 2014 | 10 | 6 | 60 | 6 | 60 |
| 2015 | 140 | 57 | 40.7 | 90 | 64.3 |
| 2016 | 101 | 41 | 40.6 | 74 | 73.3 |
| 2017 | 130 | 54 | 41.5 | 100 | 76.9 |
| 2018 | 161 | 74 | 46 | 113 | 70.2 |
| 2019 | 125 | 67 | 53.6 | 84 | 67.2 |
| 2020 | 27 | 5 | 18.5 | 10 | 37 |
| 2021 | 54 | 2 | 3.7 | 16 | 29.6 |
| 2022 | 19 | 5 | 26.3 | 7 | 36.8 |
| 2023 | 136 | 51 | 37.5 | 77 | 56.6 |
| 2024 | 342 | 135 | 39.5 | 230 | 67.3 |
| Total | 1245 | 497 | 39.9 | 807 | 64.8 |

**Table S2. Reference sequences of the G gene from hMPV genotypes identified from Genbank**

| Subtype | Country | Strain | Yearof isolation | GenBank No. | Genotype |
| --- | --- | --- | --- | --- | --- |
| A | USA | TN82-518 | 2013 | JF929832.1 | A1 |
|  | India | 00-1/2001 | 2001 | AF371337.2 | A1 |
|  | Canada | H0203-025-A | 2003 | KF178956.1 | A1 |
|  | China | BJ35624 | 2012 | MF462403.1 | A1 |
|  | Canada | 17-2000 | 2004 | AY485234.1 | A2a |
|  | USA | TN94-49 | 2011 | JN184400.1 | A2a |
|  | Australia | 145570377 | 2003 | KC403979.1 | A2a |
|  | Canada | H0607-185-A | 2007 | KF178969.1 | A2a |
|  | Peru | CFI1296 | 2011 | KJ627388.1 | A2a |
|  | Saudi Arabia | Riyadh_131 | 2009 | KT032178.1 | A2a |
|  | Peru | CFI1212 | 2011 | KJ627382.1 | A2b |
|  | Peru | PERFLE7557 | 2009 | KJ627430.1 | A2b |
|  | Kenya | KEN-020 | 2008 | KT191303.1 | A2b |
|  | Kenya | KEN-124 | 2011 | KT191350.1 | A2b |
|  | Malaysia | MYU2776 | 2013 | KU320920.1 | A2b |
|  | Japan | P7564 | 2014 | LC192174.1 | A2b |
|  | China | SA201901972019 | 2019 | MN944086.1 | A2b |
|  | Netherlands | A10 | 2010 | JN200816.1 | A2c |
|  | China | CQ1244 | 2008 | JX082176.1 | A2c |
|  | China | CQ1877 | 2010 | JX082189.1 | A2c |
|  | Malaysia | U2151 | 2013 | KU320914.1 | A2c |
|  | USA | NM005 | 2016 | KY474535.1 | A2c |
|  | China | SA20180217 | 2018 | MN944071.1 | A2c |
|  | Netherlands | NL/6/17 | 2017 | OL794455.1 | A2c180nt-dup |
|  | Australia | WM2590696 | 2017 | MW221989.1 | A2c180nt-dup |
|  | Japan | P8462 | 2016 | LC192252.1 | A2c180nt-dup |
|  | Spain | NSVH2016-09-77020 | 2016 | KX829103.1 | A2c180nt-dup |
|  | Japan | P7406 | 2014 | LC192239.1 | A2c180nt-dup |
|  | Japan | P7820 | 2015 | LC192243.1 | A2c180nt-dup |
|  | Spain | NSVH2015-19-63118 | 2015 | KX829081.1 | A2c180nt-dup |
|  | China | BJ73485 | 2016 | MF462419.1 | A2c180nt-dup |
|  | Croatia | HR129-17 | 2017 | MK947192.1 | A2c180nt-dup |
|  | China | SA20180012 | 2018 | MN944056.1 | A2c180nt-dup |
|  | Netherlands | NL/7/16 | 2016 | OL794465.1 | A2c180nt-dup |
|  | Japan | P9224 | 2017 | LC360498.1 | A2c111nt-dup |
|  | Japan | P9767 | 2019 | LC466057.1 | A2c111nt-dup |
|  | Japan | SGH-23 | 2018 | LC671555.1 | A2c111nt-dup |
|  | Japan | H585 | 2019 | LC769209.1 | A2c111nt-dup |
|  | China | 18JB00311 | 2018 | MK087726.1 | A2c111nt-dup |
|  | China | P17072 | 2017 | MK450165.1 | A2c111nt-dup |
|  | Crotia | HR761-17 | 2017 | MK947194.1 | A2c111nt-dup |
|  | China | SA20180175 | 2018 | MN944066.1 | A2c111nt-dup |
|  | China | SA20190137 | 2019 | MN944076.1 | A2c111nt-dup |
|  | China | bj2720 | 2019 | MZ851794.1 | A2c111nt-dup |
|  | China | bj1367 | 2018 | MZ851993.1 | A2c111nt-dup |
|  | China | bj0154 | 2017 | MN745086 | A2c111nt-dup |
|  | China | 2019HDHXD-389 | 2019 | OL625613.1 | A2c111nt-dup |
|  | China | 2018HDHXD-368 | 2018 | OL625628.1 | A2c111nt-dup |
| B | USA | TN982-42 | 1998 | JF929862.1 | B1 |
|  | Australia | 159148534 | 2004 | KC562230.1 | B1 |
|  | USA | C2-202 | 2004 | KC562235.1 | B1 |
|  | Canada | C0910-1014 | 2010 | KF179043.1 | B1 |
|  | Iran | 110 | 2015 | KU176106.1 | B1 |
|  | Japan | P6865 | 2013 | LC192204.1 | B1 |
|  | Japan | P8439 | 2016 | LC192213.1 | B1 |
|  | Japan | P8907 | 2017 | LC337929.1 | B1 |
|  | Japan | SGH-20 | 2017 | LC671554.1 | B1 |
|  | Japan | O53 | 2018 | LC769210.1 | B1 |
|  | Japan | OR677 | 2022 | LC769218.1 | B1 |
|  | China | bj0123 | 2018 | MK820375.1 | B1 |
|  | China | SA20180160 | 2018 | MN944088.1 | B1 |
|  | China | SA20190237 | 2019 | MN944091.1 | B1 |
|  | China | BJ831 | 2011 | MZ504959.1 | B1 |
|  | China | BJ3837 | 2013 | MZ504963.1 | B1 |
|  | China | bj0102 | 2017 | ON168639.1 | B1 |
|  | China | BJ4879 | 2005 | DQ270221.1 | B1 |
|  | Japan | P6788 | 2013 | LC192214.1 | B2 |
|  | Japan | P8429 | 2016 | LC192238.1 | B2 |
|  | Japan | SGH-30 | 2018 | LC671557.1 | B2 |
|  | Nepral | 6285-B1 | 2013 | MK179443.1 | B2 |
|  | Kenya | 02/KEN | 2012 | MK588637.1 | B2 |
|  | China | SA20190229 | 2019 | MN944096.1 | B2 |
|  | China | BJ5980 | 2014 | MZ504964.1 | B2 |
|  | China | BJ8088 | 2015 | MZ504966.1 | B2 |
|  | China | 2019HDHXD-244 | 2019 | OL625641.1 | B2 |
|  | Netherlands | NL/5/13 | 2013 | OL794439.1 | B2 |
|  | Netherlands | NL/7/17 | 2017 | OL794466.1 | B2 |

**Table S3. Reference sequences of the F gene from hMPV genotypes identified from Genbank**

| Subtype | Country | Strain | Year of isolation | GenBank No. | Genotype |
| --- | --- | --- | --- | --- | --- |
| A | Australia | 145371295 | 2003 | KC403977.1 | A1 |
|  | China | BJ-1610 | 2012 | KU821121.1 | A1 |
|  | Netherlands | 00-1 | 2001 | AF371337.2 | A1 |
|  | USA | TN96-1 | 1999 | JN184399.1 | A1 |
|  | Japan | JP-03-180 | 2004 | AY530092.1 | A1 |
|  | USA | TN-82-518 | 1982 | KC403973.1 | A1 |
|  | Peru | CFI1717 | 2012 | KJ627428.1_ | A2a |
|  | Japan | Jpn03-1 | 2010 | AB503857.1 | A2a |
|  | Canada | CAN00-16 | 2002 | AY145301.1 | A2a |
|  | Netherlands | NL/17/00 | 2003 | AY304360.1 | A2a |
|  | Australia | 145570377 | 2003 | KC403979.1 | A2a |
|  | Argentina | ARG/107 | 2002 | KF686742.1 | A2a |
|  | Thailand | 45 | 2011 | JQ745068.1 | A2a |
|  | Netherlands | NL/00/17 | 2000 | FJ168779.1 | A2a |
|  | USA | C1-718 | 2005 | KC562220.1 | A2b |
|  | Peru | CFI1212 | 2011 | KJ627382.1 | A2b |
|  | Japan | SGH-36 | 2018 | LC671558.1 | A2b |
|  | Japan | O962 | 2019 | LC769216.1 | A2b |
|  | China | BJ3357 | 2013 | MZ504962.1 | A2b |
|  | Netherlands | NL/10/17 | 2017 | OL794375.1 | A2b |
|  | Netherlands | NL/2/14 | 2014 | OL794401.1 | A2b |
|  | Japan | JPS03-240 | 2004 | AY530095.1 | A2b |
|  | China | BJ1887 | 2006 | DQ843659.1 | A2b |
|  | Thailand | THL/29 | 2011 | JQ745052.1 | A2b |
|  | India | NIV1113764 | 2011 | KC731515.1 | A2b |
|  | China | 2015GZ56783 | 2015 | MT253760.1 | A2b |
|  | China | GZ01 | 2009 | GQ153651.1 | A2b |
|  | Japan | SGH-23 | 2018 | LC671555.1 | A2c |
|  | USA | SC3141 | 2015 | MF045424.2 | A2c |
|  | China | 2018GZ57818 | 2018 | MT253785.1 | A2c |
|  | China | 2018GZ58642 | 2018 | MT253790.1 | A2c |
| B | Thailand | THL/42 | 2011 | JQ745065.1 | B1 |
|  | China | N39 | 2008 | GU048746.1 | B1 |
|  | Australia | 159148534 | 2004 | KC562230.1 | B1 |
|  | China | bj0123 | 2018 | MK820375.1 | B1 |
|  | India | NIV113714 | 2011 | KC731523.1 | B1 |
|  | Croatia | HR124-12 | 2012 | KU375616.1 | B1 |
|  | Netherlands | NL/1/99 | 2004 | AY525843.1 | B1 |
|  | Thailand | THL/68/2011 | B1 | JQ745091.1 | B1 |
|  | Croatia | HR1659-13 | 2013 | KU375620.1 | B1 |
|  | China | 2011GZ01427 | 2011 | MT107173.1 | B1 |
|  | USA | 2019_2910 | 2019 | MT118718.1 | B1 |
|  | China | BJ1816 | 2006 | DQ843658.1 | B2 |
|  | Croatia | HR89-12 | 2012 | KU375614.1 | B2 |
|  | USA | SC3259 | 2015 | MF045425.1 | B2 |
|  | China | 05641 | 2014 | MT253756.1 | B2 |
|  | Japan | I1027 | 2016 | LC337751.1 | B2 |

**Table S4. The number of reads and the GenBank accession numbers of 20 hMPV whole genomes**

| Sample No. | GenBank No. | Reads | Read length | |
| --- | --- | --- | --- | --- |
|  |  |  | R1 | R2 |
| 2024-02R3820 | PV218056 | 2,921,564 | 1-150 | 1-150 |
| 2024-01R2925 | PV218062 | 3,824,686 | 1-150 | 2-150 |
| 2024-01R2969 | PV218063 | 3,584,029 | 1-150 | 1-150 |
| 2024-00R1049 | PV218067 | 3897608 | 1-150 | 1-150 |
| 2024-12R0597 | PV218069 | 3209128 | 1-150 | 1-150 |
| 2024-00R1081 | PV218072 | 5822206 | 2-150 | 1-150 |
| 2024-15R0346 | PV218058 | 4553722 | 1-150 | 1-150 |
| 2024-02R4253 | PV218099 | 3255764 | 1-150 | 1-150 |
| 2024-03R1379 | PV218100 | 5037312 | 1-150 | 1-150 |
| 2024-09R0361 | PV218102 | 5365968 | 4-150 | 3-150 |
| 2024-11R0451 | PV218103 | 3993000 | 1-150 | 1-150 |
| 2024-13R0395 | PV218104 | 3530890 | 1-150 | 1-150 |
| 2024-15R0417 | PV218106 | 4296620 | 1-150 | 1-150 |
| 2024-R0373 | PV218109 | 678520 | 1-150 | 3-150 |
| 2024-04R1517 | PV218111 | 5908678 | 4-150 | 1-150 |
| 2024-04R1531 | PV218114 | 2457328 | 1-150 | 2-150 |
| 2024-04R0576 | PV081138 | 4,173,514 | 1-150 | 1-150 |
| 2024-01R1679 | PV081139 | 2,007,064 | 1-150 | 1-150 |
| 2024-11R0422 | PV081140 | 3,797,280 | 1-150 | 1-150 |
| 2024-01R3091 | PV081141 | 7,616,784 | 1-150 | 1-150 |
